# Caries of the Superior Maxilla

**Published:** 1881-02

**Authors:** Truman W. Brophy

**Affiliations:** Chicago.


### ARTICLE V.

### *Caries of the Superior Maxilla.*

BY TRUMAN W. BROPHY, M. D., D. D. S , CHICAGO.

On October 26th, 1879, I was called in consultation with the family physician of the patient whose case I now report.

A lady aged thirty-five, somewhat anæmic, had an abscess form about two years previously, in the canine fossa of the left superior maxilla. It was lanced by her physician at a point just below the orbit and to the right of the infra-orbital foramen. It discharged freely. The wound, however, refused to heal, and there was constantly a discharge of sero-pus which excoriated the skin to an extent corresponding in size with a silver half-dollar. Having treated the patient several months without improvement, her physician directed her to a surgeon, under whose treatment she remained about a year. This treatment consisted of introducing a seton into the fistula, passing it downwards, and carrying it through the mucous membrane, at a point where it folds upon itself (the gingevo-labial groove.) Besides this, injections of solutions of carbolic acid were made. This treatment was kept up faithfully during the period above mentioned, without abating the disease in the least.

Indeed, the patient was gradually becoming weaker and despondent. Losing faith in her medical attendant, she drifted, as patients frequently do, from one practitioner to another, until she had employed several. The disease had been pronounced cancer by some physicians, who prognosticated death within two years. When she came under the care of the gentleman with whom I saw her, she was declining very fast. Under his treatment, however, her health improved. There existed in her case a general catarrhal condition.

The patient had, during the progress of the disease, been under the care of a practitioner of dentistry, who had skil fully filled the six anterior superior teeth, which were carious on the labial surfaces about the margins of the gums. The teeth were not carious to that extent, however, which would expose their pulps, or necessarily endanger their vitality. Neither was there any discoloration or other objective symptoms of devitalized pulps. The condition of the case was such as most frequently results from alveolar abscess, which cannot occur without the death of the tooth pulp. Examining the face carefully, I found that a probe passed into the fistula, came in contact with denuded bone. Believing that the inital lesion could be traced to the teeth, and not having any instruments with me with which to test their vitality, I improvised the following method, which served the purpose admirably. With a small round file heated, I ascertained by applying it to the gold fillings (had there been no fillings, I would have applied it to the teeth,) that the left canine tooth was, unlike the others, devoid of sensation: and it was at the apex of the fang of this tooth where caries of the bone was found. This enabled me to make a diagnosis of the case.

The patient accompanied me to my office, where I verified my diagnosis by drilling through the palatal surface of the non-sensitive canine tooth, when I found, as I had anticipated, the pulp canal filled with sero-pus, and by introducing the point of a syringe into the cavity in the tooth, liquid was forced through the pulp canal into the abscess and out of the fistulous opening upon the cheek. Having thus demonstrated that the diagnosis was correct, I decided at once upon the course to pursue in effecting a cure. With a bistoury, I made an incision through the mucous membrane, down upon the apex of the affected tooth, and into the cavity formed by the destruction of the bone. After controlling the hæmorrhage, by the use of a nasal speculum a clear view of the abnormal parts was secured. The alveolar process had become carious, to that extent, which exposed about one-third of the upper portion of the root of the canine tooth above mentioned. With an engine drill, I cut away the exposed portion of the root, and the carious bone surrounding it, then filled the canal with gutta percha, and smoothed it off carefully at the apex. This done, the cavity and wound were filled with boracic acid crystals. This treatment, together with general tonics, was continued, keeping up free drainage, until the cavity left by the destruction of the bone was completely filled with granulations, when the wound was permitted to heal. The fistula upon the cheek healed as soon as drainage was secured from the lowest portion of the sac. The practice, so frequently resorted to, I regret to say, of lancing alveolar abscesses externally, cannot be too vigorously condemned. Unsightly scars are thus made, all of which might be avoided by opening the abscesses within the mouth.

Three months after the operation I filled permanently, with gold, the cavity made to reach the pulp canal, which completed my services to the patient. This case was of traumatic origin. Prior to the development of the disease, the patient had received a blow, cutting the lip quite severely, on the affected side, and it was due to this accident, which severed the vessels and nerves of the pulp of the canine tooth, that its vitality was destroyed.

After the pulp became devoid of vitality, suppuration and generation of gases followed in close succession, when the pus was forced through the apical foramen, with the result above given.

The question would naturally be asked, what do you expect of a tooth after the loss of its pulp? Is it not then a foreign substance, and therefore incompatible with the economy? My answer would be, No. Certain tissues of the tooth, the enamel and dentine, have lost their source of nourishment. The cementum, however, which forms the external portion of the root, closely resembles true bone, like which it contains lacunæ and canaliculi; and is nourished by the vessels of the periosteum. Therefore, we have an organ not wholly devoid of vitality, and when properly treated, will serve for many years the purpose for which it was intended.

I have no doubt that a very large percentage of the cases of caries and necrosis of the maxillary bones, as well as trigeminal neuralgias, with all their complications, emanate from lesions of the dental organs. And it is not too much to say that if, instead of resorting to their indiscriminate removal, they were treated upon scientific principles, as other diseases are, the requirements of humanity would be far more effectually subserved.

---

